# Pearl Microstructure and Expression of Shell Matrix Protein Genes MSI31 and MSI60 in the Pearl Sac Epithelium of *Pinctada fucata* by *In Situ* Hybridization

**DOI:** 10.1371/journal.pone.0052372

**Published:** 2013-01-14

**Authors:** Yu Sato, Nariaki Inoue, Takashi Ishikawa, Ryo Ishibashi, Mayu Obata, Hideo Aoki, Takashi Atsumi, Akira Komaru

**Affiliations:** 1 Mie University, Graduate School of Bioresources, Tsu, Mie, Japan; 2 Mie Prefecture Fisheries Research Laboratory, Shima, Mie, Japan; University of Connecticut, United States of America

## Abstract

Expression patterns of the shell matrix protein genes MSI31 and MSI60 in the pearl sac epithelium were examined by in situ hybridization 38 days after implantation, and related to pearl quality. A pearl sac that produced a nacreous pearl showed very weak expression of MSI31 and strong expression of MSI60. A pearl sac, which yielded a prismatic pearl, strongly expressed MSI31 and very weakly expressed MSI60. In a complex pearl, whose surface consisted of a mosaic of both nacreous and prismatic layers, the expression pattern of MSI31 and MSI60 similarly corresponded to the underlying surface structures of the pearl. A nacreous pearl whose pearl sac showed strong MSI31 expression had an entirely nacreous surface composed of a laminar structure with unusual tablet growth at the corresponding site. MSI31 and MSI60 are the major components of the shell matrix proteins of the nacreous and prismatic layers. Clearly, high expression of MSI31 does not always result in prismatic secretion. These observations cannot be explained solely on the basis of the expression patterns of MSI31 and MSI60. We propose that, in addition to the MSI genes that form the prismatic and nacreous layers, upstream from these genes there are regulatory master genes that determine whether a nacreous layer (aragonite) or a prismatic layer (calcite) is formed.

## Introduction

Immediately after the implantation of a nucleus prepared from a freshwater mussel shell and a small piece of mantle tissue from a donor oyster, into a recipient pearl oyster *Pinctada fucata*, the outer epithelium from the mantle graft migrates over the surface of the nucleus and completely envelopes it [1]. This epithelial tissue around the nucleus is termed the pearl sac. The pearl sac epithelium starts to secrete the shell components onto the surface of the nucleus [2], [3]. Normally, during the early stages of pearl formation following implantation, the periostracum is secreted by the epithelium of the pearl sac onto the pearl surface. Then, the prismatic layer and, finally, the nacreous layer are formed in sequence [4].

The thickness of the prismatic layer determines the quality of pearls; those with a thick periostracum and prismatic layer are considered to be of rather low quality [5], [6], [7]. Occasionally, the nacreous layer is formed directly onto the periostracum [8]. In cross-sections of high quality nacreous pearls, the prismatic layer appears as a very thin concentric circle [7]. This suggests that the epithelial cells switched from prismatic to nacreous secretion almost simultaneously, even though secretion of the underlying nacreous layer commenced over a more extended period and did not proceed evenly over the surface of the pearl [9]. Elucidation of changes in the expression patterns of genes for prismatic and nacreous layer formation during the early stage of pearl formation would be valuable for the control the pearl quality.

The previous extensive studies on the shell matrix proteins in *Pinctada fucata* have been reviewed [10], [11]. The proteins MSI31 [12], MSI7 [13], Shematrin [14], Prismalin-14 [15], Prismin [16], Prisilkin-39 [17], and Aspein [18] are considered to be specific framework proteins of the prismatic layer. Nacreous layer shell matrix genes, including MSI60 [12], N16 [19], and Pearlin [20] have also been characterized. ESTs in *Pinctada fucata* mantle tissue and pearl sac have been analyzed and screened for novel candidates related to shell formation [21]. Such studies will accelerate clarification of the whole mechanism of pearl formation.

These studies demonstrated expression patterns of MSI31 and MSI60 in the mantle epithelium by *in situ* hybridization (ISH) [12]. The expression patterns of genes have also been examined using real time PCR, including MSI31, MSI60, Aspein, and Prismalin, [22]. An ISH study [12] showed restricted expression of the prismatic layer genes MSI31, Prismalin-14, and Aspein in the ventral region of the mantle (the mantle edge). In contrast, real time PCR indicated that nacreous formation genes, such as MSI60 and N16, were expressed only in the dorsal (pallial) region of the mantle. It is possible that the distinct expression patterns of the genes in the mantle tissue that form the prismatic and nacreous layers are regulated by genes similar to the compartment selector genes described in *Drosophila* [22].

Using real time PCR, a correlation was reported between the quality of pearls and gene expression patterns in the pearl sac epithelium [23]. However, although real time PCR is able to estimate very accurately the expression levels of shell matrix genes, it is cannot be used to distinguish local expression patterns in the pearl sac epithelium.

The present study uses ISH to compare the expression patterns of MSI31 and MSI60, as representative genes of the shell matrix framework, in the pearl sac epithelium of high and low quality pearls. We prepared ISH probes to detect mRNAs of MSI31 and MSI60 and revealed their expression patterns in the pearl sac epithelium at an early stage of pearl formation. In particular, we compared the gene expression patterns corresponding to pigmentation and flaws on the pearl with their surface microstructures observed by scanning electron microscopy (SEM).

## Results

### *In situ* hybridization of juveniles

**Figure 1:**
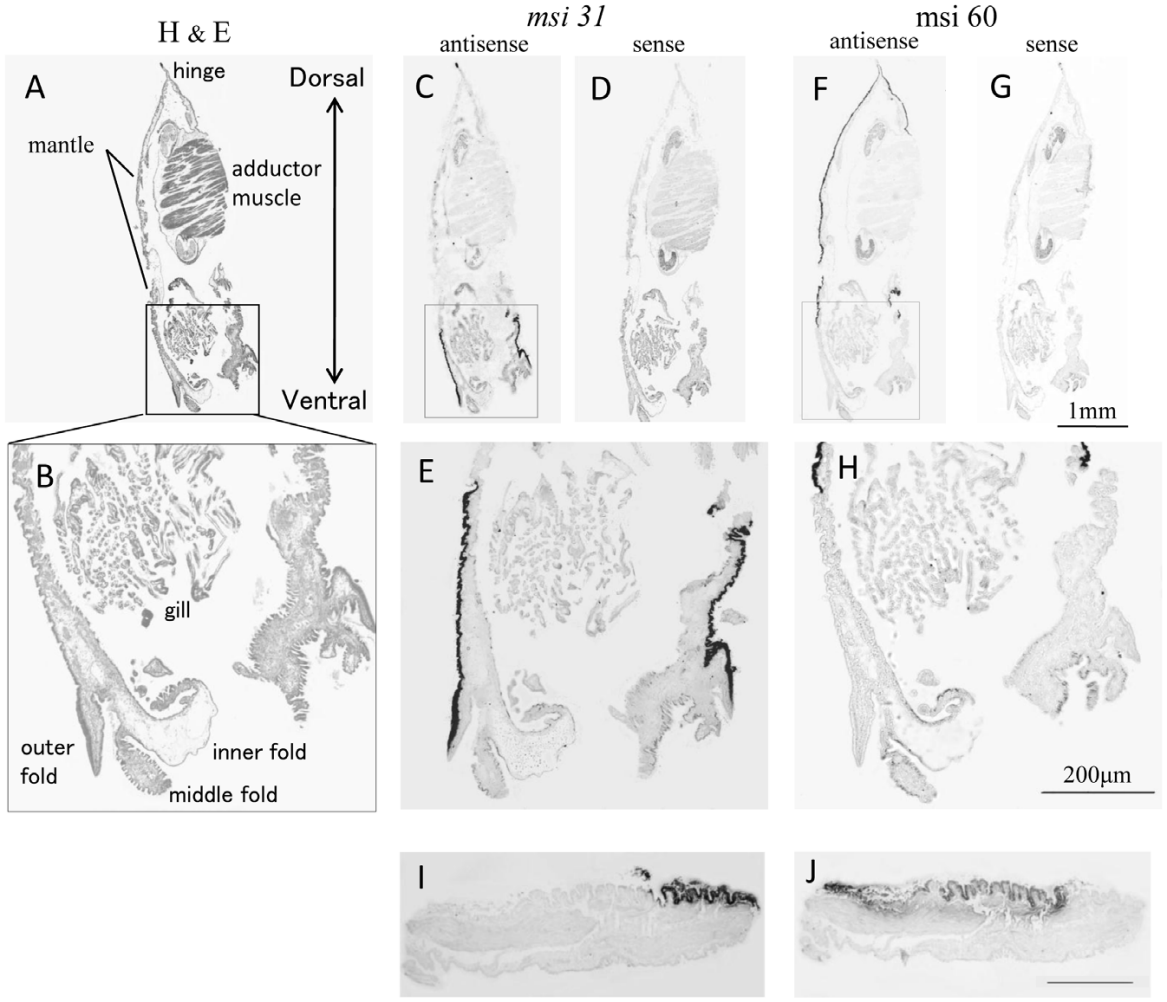
Transverse section of a whole juvenile oyster stained by H & E and MSI probes. (A, B) H & E, (C, E) MSI31 probe; (F, G, H) MSI60 probe. B, E, and H are enlarged images of the areas enclosed by boxes in A, C, and F. (I, J) A piece of mantle for implantation stained by MSI31 (I) and MSI60 (J) probes.

Fig. 1 shows transverse sections of whole juveniles stained with H&E (A, B), MSI31 anti-sense probe (C, E), MSI31sense probe (D), MSI60 anti-sense probe (F, H), and MSI60 sense probe (G). MSI31 was expressed in the ventral region of the epithelium on the outside of the mantle tissue (Fig. 1C). At the outer fold, only the outer surface of the epithelium (the side facing the shell) showed MSI31 expression (Fig. 1E). Expression of MSI60 was detected in the outer mantle epithelium from the dorsal to the ventral regions of the mantle (Fig. 1F, H). No MSI60 signal was detected in the epithelium at the mantle edge. There was no overlap in the expression of MSI31 and MSI60 in the mantle tissue.

### Mantle implants

Small pieces of mantle prepared for implantation were transversely sectioned in a plane parallel to the dorsal and ventral axis. MSI31 and MSI60 were expressed only in the outer epithelium facing the shell. The ventral region (marginal area) of the epithelium expressed MSI31 (Fig. 1I) and the dorsal region showed MSI60 expression (Fig. 1J). The expression patterns of both genes had distinct boundaries with the border between them lying close to the ventral edge. Three pieces, taken from a single individual, showed similar expression pattern.

### Pearl quality

**Figure 2:**
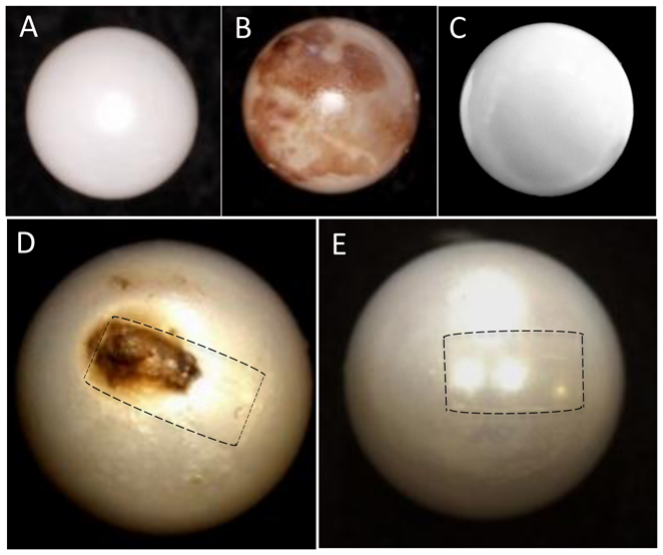
General images of the pearls examined in the present study. (A) nacreous pearl; (B) prismatic pearl; (C) pearl without deposition; (D) complex pearl with a flaw and pigmentation; (E) nacreous pearl with unusual tablet formation. The areas shown by broken lines in D and E correspond to the pearl sac epithelium shown in Figs. 4A and 6A, respectively.

Based on observation of their surface microstructures by SEM, we selected the following nine pearls for in situ hybridization of their pearl sacs (Fig. 2): nacreous pearls (n = 2) in which the surface of the pearl consisted of only a nacreous layer (Fig. 2A); prismatic pearls (n = 1) whose surface was covered by only a prismatic layer (Fig. 2B); a pearl without deposition whose surface was in a similar condition to that before implantation (n = 1; a single oyster was obtained with a pearl in this condition) (Fig. 2C); complex pearls (n = 2), whose surface consisted of nacreous and prismatic layers with pigmentation and flaws (Fig. 2D); a nacreous pearl with unusual nacre tablet growth (n = 1; a single pearl was encountered in this condition). The surface of the latter pearl exhibited a complete nacreous layer and it was graded by eye as high a quality nacreous pearl. However, SEM observation revealed atypical nacreous tablet formation in some regions (Fig. 2E).

### Nacreous pearl (n = 2)

**Figure 3:**
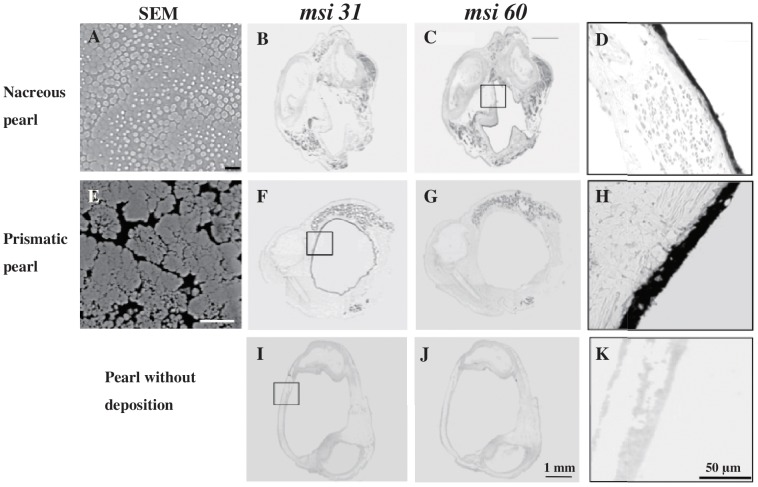
Surface microstructure of pearls observed in the scanning electron microscope (SEM), and gene expression in the pearl sac detected by ISH. (A, E) SEM images of a nacreous pearl and a prismatic pearl. (B, F, I) MSI31 and (C, G, J) MSI60 gene expression in the pearl sac epithelium. D, H, and K) are enlarged images of the regions outlined in C, G, and J, respectively. (I, J, K) MSI31 and MSI60 expression patterns in the pearl sac of the oyster which produced the pearl without deposition. The grade of the pearls was determined by eye and by scanning electron microscopy.

Fig. 3A shows an SEM image of a nacreous pearl without flaws or pigmentation. The surface of the pearl consisted of nacreous layers only. Flat hexagonal tablets were deposited on the surface of the pearl in a regular contiguous pattern, forming a laminar structure. Newly formed crystals on the surface of the tablets were small and round and less than 1 µm in diameter. Fully-developed tablets were hexagonal and approximately 5–6 µm in diameter.

Because of shrinkage during the embedding, the pearl sac did not appear circular in cross-section. MSI31 expression was not observed in the pearl sac (Fig. 3B) but the strong MSI60 signal was clearly recognizable along the epithelial margin of the pearl sac (Fig. 3C, D).

Two oysters showed similar expression patterns.

### Prismatic pearl (n = 1)

The surface of this type of pearl consisted of only a dark prismatic-like layer (Fig. 3E). SEM micrographs showed irregular polygonal crystals, 1–10 µm in diameter, deposited on the dark matrix. These crystals were needle-like crystals with their axes perpendicular to the surface of the pearl. The surface of the prismatic pearls appeared different from that of the prismatic layer of the shell and we were unable to observe the interprismatic wall in these pearls.

The whole of the pearl sac epithelium showed a strong MSI31 signal (Fig. 3F, H), but no expression of MSI60 was detected (Fig. 3G) in three oysters.

### Pearl without deposition (n = 1)

Although a pearl sac was formed around the nucleus, no deposition of the organic matrix, or of the prismatic and nacreous layers, was observed on the surface of the nucleus, even by SEM. Likewise, we were unable to detect any expression signal of either MSI31 (Fig. 3I, K) or of MSI60 (Fig. 3J).

Table 1 summarizes the expression pattern in the pearl sac epithelium of the pearls described above.

**Table 1:** Summary of expression levels of MSI 31 and MSI 60 by ISH in the pearl sac epithelium.

pearl category	MSI 31	MSI 60
nacreous pearl (n = 2)	−	+++
prismatic pearl (n = 1)	+++	−
pearl without deposition (n = 1)	−	−
complex pearl (Fig. 4, n = 1)		
nacreous portion	−	+++
prismatic portion	+++	−
complex pearl (Fig. 6, n = 1)		
nacreous portion (normal)	−∼+++	+++
nacreous portion (unusual)	+++∼+	−

### Complex pearl with flaw and dark pigmentation (n = 2)

**Figure 4:**
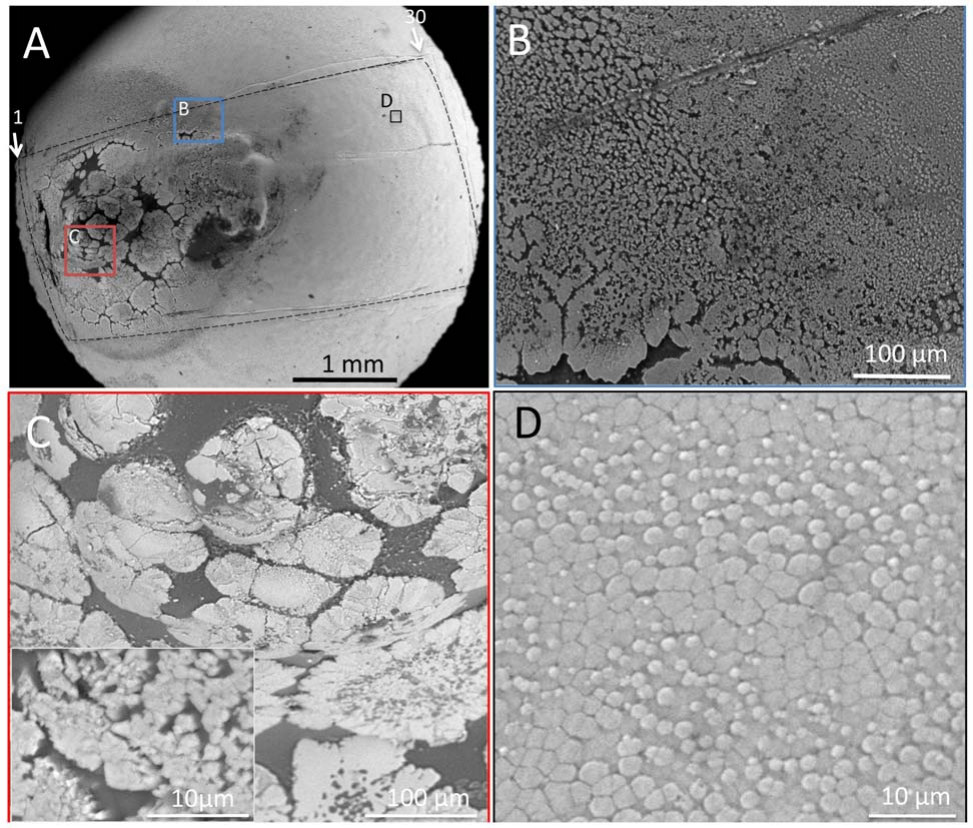
SEM images of a complex pearl with dark pigmentation and a flaw. (A) Low power SEM image. The line (1, 30) and arrows indicate the plane of the sections of the pearl sac shown in Fig. 5. (B) The boundary region between the dark pigmentation site and the nacreous layer. The blue, red, and black boxes indicate the areas shown at higher magnification in panels B, C and D. The broken line outlines the area corresponding to pearl sac tissue examined by ISH and shown in Fig. 5. (C) Enlarged image of the protruded area shown by the red box in A. Inset shows the image at higher magnification. (D) Enlarged image of the nacreous layer shown as box D in Fig. 5A.

The two pearl oysters that yielded complex pearls with flaws showed essentially similar MSI expression pattern in the pearl sac epithelium. Images from one of the oysters are shown. This pearl exhibited dark pigmentation and a protrusion on the surface (Fig. 4A, B). Fig. 4C shows an enlargement of the boundary area between the pearl layer and the pigmentation site. Nacreous layers were deposited in the region shown at the right-hand side of Fig. 4B. Near the flaw, the surface of the dark matrix was covered by rosette-like prisms (Fig. 4C) similar to those observed by Wada [8] and composed of a bundles of fine needle-like crystals arranged perpendicularly to the surface (Fig. 4C, inset). The interprismatic wall in the shell prismatic layer was not observed. These crystals were present on the dark matrix and occasionally on the dark granular substance. Typical nacreous tablets were deposited in non-flawed areas in a laminar arrangement (Fig. 4D). The small tablets were less than 1 µm in diameter, round, and deposited in the typical pavement-like arrangement observed in nacreous pearls.

### Expression of MSI31 and MSI60 in the pearl sac epithelium of the complex pearl

**Figure 5:**
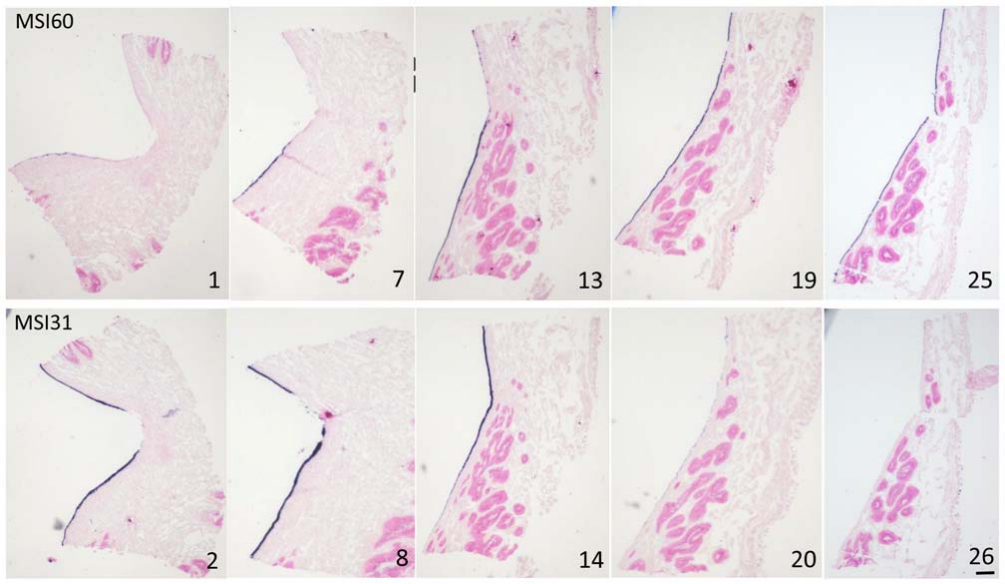
Expression of MSI31 and MSI60 in pearl sac epithelium detected by ISH. This oyster produced the pearl with pigmentation and a flaw (protrusion) shown in Fig. 4. Images in the upper and lower rows show the expression of MSI60 and MSI31, respectively. The numbers refer to sequence of serial sections. Sections 1 and 30 corresponded to the plane indicated by arrows on Fig. 4A (sections 27–30 are not shown). Scale bar is100 µm.

Fig. 5 shows the expression patterns of MSI31 and MSI60 in representative sections of a complex pearl, as detected by ISH. The pearl sac on the flaw appeared V-shaped, corresponding to the shape of the flaw The pearl sac epithelium corresponding the prismatic layer on the flaw showed strong expression of MSI31 (Fig. 5, sections 2 and 8). In contrast, the expression of MSI60 was rather weak in this region (Fig. 5, sections 1 and 7). The epithelium corresponding to the boundary area between the prismatic and nacreous layers exhibited moderate expression of both MSI31 and MSI60. In the area of the pearl sac corresponding to the pearl layer, MSI31 showed very weak intensity (Fig. 5, sections 20 and 26) and MSI60 showed strong intensity (Fig. 5, sections 19 and 25).

### SEM observation of a nacreous pearl with unusual nacreous tablet growth (n = 1)

**Figure 6:**
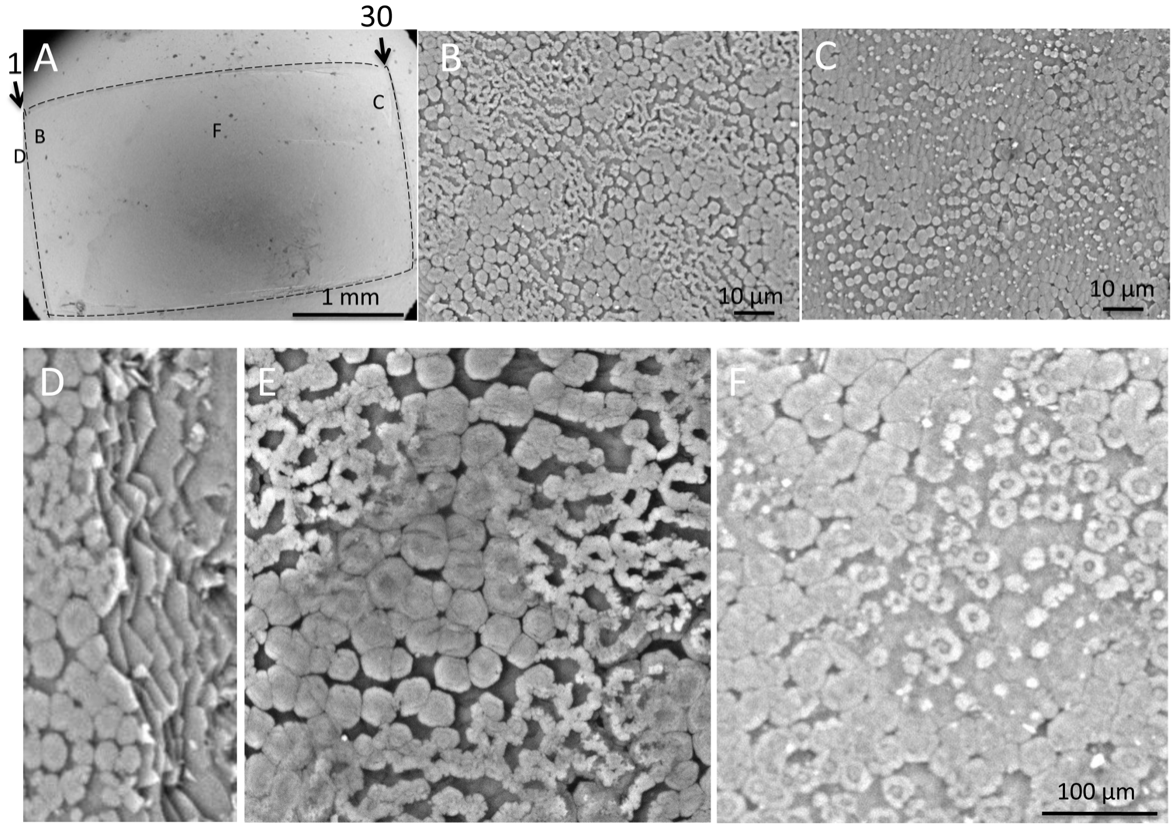
SEM images of the nacreous pearl with unusual tablet growth. (A) The surface of the pearl at low magnification. The numbers and arrows indicate the serial section sequence and the sectioning plane of the pearl sac tissue stained by ISH referred to in Fig. 8. (B) SEM micrograph of the surface near point B in panel A. The process of tablet growth was different from a typical nacreous pearl shown in Fig. 3A. (C) Enlarged image of the pearl surface around point C in panel A. (D) A partial cross-section showing the lamellar structure beside the scratch. (E) The surface structure of B shown at higher magnification. Note doughnut-shaped structures. (F) Ring like structure of the growing nacreous tablets between points B and C in panel A. Scale bars are 1 mm in A, 10 µm in B and C, and 100 µm in D–F.

To the naked eye, the outward appearance of this pearl, which lacked pigmentation or flaws, was similar to the nacreous pearl (Figs. 2E and 6A). However, SEM observation revealed an area where the newly deposited tablets exhibited an unusual doughnut or honeycomb-like shape (left-hand side of the area shown by the broken line in Fig. 6A). Elsewhere, the grown crystals exhibited the typical structure of the nacreous layer (Fig. 6C), as observed in nacreous pearls (Fig. 3A). Fig. 6D shows a partial cross-section along a scratch with a scalpel near point B, revealing the underlying laminar structure. The fully-grown tablets, 3–5 µm in diameter, formed the laminar structure typical of the nacreous layer. However, in the growing tablets concentric rings (Fig. 6E) were observed, as shown at the left-hand side. Interestingly, the newly deposited tablets formed the doughnut or honeycomb like structure (Fig. 6E). As shown in Fig. 6F, a small core was present inside the ring-like structure. These features differed from those of the typical nacreous pearl shown in Fig. 3A, and of the nacreous layers of the shell.

### Expression of MSI31 and MSI 60 in the pearl sac epithelium of the unusual nacreous pearl

**Figure 7:**
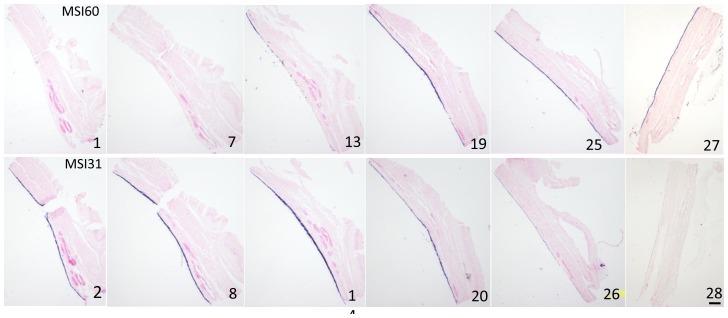
Expression of MSI31 and MSI60 in pearl sac epithelium detected by ISH in a nacreous pearl with unusual tablet growth. This oyster produced the pearl shown in Fig. 6A. Upper and lower rows show the expression of MSI31 and MSI60, respectively. The numbers refer to the sequence of serial sections. Section 1 and 30 correspond to the plane indicated by arrows in Fig. 6A. Sections 29–30 are not shown.

Fig. 7 shows representative sections of the pearl sac epithelium stained by the MSI31 or MSI60 probes. The area of the pearl sac epithelium to the left in Fig. 7 (sections 2–14) showed strong expression of MSI31 but expression of MSI60 was not detectable. In contrast, MSI60 was strongly expressed on the right-hand side in Fig. 7 (sections 19–27) but MSI31 expression was weak (Fig. 7, sections 26 and 28).

**Figure 8:**
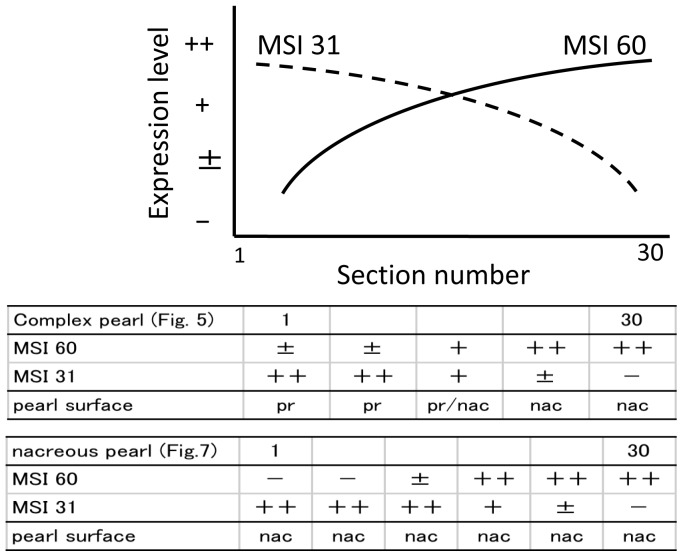
Schematic expression pattern of MSI31 and MSI60 based on the observations of the pearl sac of a complex pearl and a nacreous pearl with unusual tablet growth and the corresponding pearl surface observed in the SEM. Nac and Pr refer to nacreous and prismatic layers, respectively. The intensity of the ISH signal is indicated: − undetectable; ± very weak; + positive; + + very strong. The numbers indicate the position of pearl sac sections.

Fig. 8 summarizes the MSI expression and corresponding surface structures of the pearl sac of the complex pearl and the nacreous pearl with unusual tablet growth. Similar patterns of expression of MSI31 and MSI60 were observed in both types of pearl, but the surface structures of the pearls were markedly different (Figs. 4 and 6).

## Discussion

### Expression of MSI31 and MSI60 in juveniles

In the juvenile, MSI31 expression was observed in the inner epithelium of the mantle edge and MSI60 expression was observed in the pallial mantle [12] by ISH. MSI60 was continuously expressed over the outer surface of mantle epithelium from the dorsal end, near the hinge, to the ventral pallial mantle. No difference in signal intensity between the pallial mantle and the dorsal end was detected and there was no overlap in the locations of expression of MSI31 and MSI60. This pattern corresponds to the two-layered shell structure consisting of an outer prismatic layer and an inner nacreous layer. The prismatic layer is produced first, and the nacreous layer is then deposited onto the prismatic layer as the oyster grows [24]. The clear boundary between MSI31 and MSI60 expression suggests that the secretion and crystallization of the shell layers are rather local events. The pearl layer is formed just proximal to the epithelium secreting MSI60. The extrapallial space (EPS) between the shell and mantle containing extrapallial fluid is a site of biomineralization and provides a common environment for the formation of both the prismatic and nacreous layers. During rapid shell growth, the mantle and shell are in very close contact at the shell edge so that the transport of ions and organic substances could occur by direct contact [25]. The clear boundary in shell structure and the discontinuous expression pattern in the mantle epithelium suggest that the formation of prismatic and nacreous layers correspond closely to epithelia expressing MSI31 and MSI60, respectively.

### Mantle graft and pearl sac epithelium

Just after dissection, the mantle graft included epithelial cells expressing MSI31 or MSI60 (Fig. 1I, J). After implantation, the epithelium proliferates and migrates from the edge of the mantle graft until it completely surrounds the nucleus [4], indicating that the pearl sac epithelium is derived from epithelium cells which had previously expressed both MSI31 and MSI60. Our previous study using real time PCR [26] showed that the expression of genes in the pearl sac epithelium initially decreased markedly and almost no transcription occurred until ten days after implantation when transcription of the shell matrix genes commenced. Once the pearl sac is formed, the expression of all genes in the pearl sac epithelium maybe regulated by common factors in spite of the different gene expression pattern just before implantation. Kawakami suggested [4] that that the process of shell matrix protein secretion is not genetically determined. This means that, after the formation of the pearl sac, genes of the host oyster must regulate the pearl sac epithelium. Our present ISH study on nacreous and prismatic pearls supports this hypothesis. In the oyster which produced the high quality nacreous pearl, the transition from prismatic layer to nacreous layer formation may have occurred simultaneously just after implantation during the early stage of pearl formation. In contrast, the oyster that produced the prismatic pearl continued to express the genes for prismatic layer formation. These processes just after implantation must be regulated by factors within the host oyster.

In the prismatic pearl, the pearl sac showed different expression patterns of MSI31 and MSI60 (Fig. 3). This may result from disturbance of the expression pattern of the shell matrix protein genes. It has been proposed that cell debris digested by hemocytes lying between the pearl and the pearl sac epithelium may cause abnormal secretion [2]. We have shown previously, by real time PCR, that the quality of pearls is correlated with MSI31 and MSI60 expression pattern in the pearl sac epithelium. The relative expression levels of MSI31 were significantly lower in the pearl sac of high quality nacreous pearls than of low quality pearls [23]. The present study using ISH showed that, in the oyster that produced the nacreous pearl, only MSI60 was detected uniformly over the epithelium of the pearl sac. In contrast, in the oyster that produced the prismatic pearl only MSI31 was detected.

In a complex pearl, MSI31 and MSI60 were differentially expressed (Fig. 5). This expression pattern of shell matrix genes presumably accounted for the complex surface structure and was consistent with the results of nacreous and prismatic pearls.

The nacreous pearl with unusual tablet growth exhibited strong MSI31 and very weak MSI60 expression in a region of the pearl sac epithelium that induced nacreous layer formation. However, the process of nacreous crystal formation was atypical (Fig. 3A). We are unable to interpret these SEM observations solely in terms of the expression patterns of MSI31 and MSI60. We suggest that the unusual pearl layer formation may represent a transitional process between prismatic layer and nacreous layer formation. At the boundary between the prismatic and nacreous layers in the shell of the *P. margaritifera* shell, fibrous aragonite was first deposited on the prismatic walls [27]. Likewise, aragonite (nacreous) crystals were deposited onto a dimple in the organic matrix of the prismatic layer at the growth front of the nacreous layer in *P. fucata* [28]. The tablets then grew concentrically. In the present study, in the unusual nacreous pearls (Fig. 6B, E), the outer ring-like framework of the tablets formed first and later the central core was occupied by crystals (Fig. 6F). This tablet growth appeared to be different between the pearls shown in Fig. 6E and Fig. 6 F, and also different from that of typical nacreous layer formation (Fig. 6C).

The presence of genes forming nacreous and prismatic layers might be controlled by different upstream regulatory factors in the mantle tissue [22]. Recently, the novel matrix proteins Pif [29] and Prisilikin-39 [17] were characterized in *P. fucata*. These proteins may have roles in the regulation of the formation of the nacreous and prismatic layers, respectively.

Our ISH observations on the unusual nacreous pearl showed that high levels of expression of MSI31 do not always result in prismatic layer formation. Our previous study using real time PCR [23] also detected the expression of MSI31 in a pearl sac which yielded a high quality nacreous pearl. We suggest that a gradient of a regulatory protein may explain nacre formation instead of the normal expression of MSI31 in the pearl. The expression of MSI60 in the region of the pearl sac epithelium that exhibited unusual nacreous tablet formation (Figs. 6B, E, and Fig. 7) was lower than observed in a previous study [30] and lower than in the nacreous pearl shown in Fig. 3A. These results indicate that the regulation of genes forming the nacreous layer by proteins in the pearl sac may not be as simple as that proposed for the mantle [22], especially at the transition just after implantation.

Although the left side of the area shown in Fig. 6A expressed MSI31 intensely, the prismatic layer was not formed in the corresponding region of the pearl sac. This implies the existence of a factor that prevented prismatic layer formation in spite of the expression of prismatic layer forming genes [23]. This putative regulatory factor may have exhibited a gradient in its level of expression in the area in Fig. 6A.

We propose that, during the transition between the formation of the prismatic and nacreous layers, an upstream regulatory gene for nacre formation overrides the MSI expression pattern; i.e., there is a master regulatory gene upstream of the MSI genes that determines whether prismatic or nacreous layers are formed. Expression of this putative master regulatory gene results in strong MSI31 and weak MSI60 expression leading to a transitional appearance of nacreous layer formation. However, MSI60 is the major protein component of the matrix of nacre. The growth of nacre tablets may be altered because of insufficient supply of MSI60 matrix protein or by disturbance to the expression of genes related to the prismatic layer. After the transition phase, the stable nacreous layer formation pattern associated with weak MSI31 and strong MSI60 and expression would be induced, as observed in typical nacreous pearls. Thus, the regulation of shell matrix genes in the pearl sac epithelium at the early stage of pearl formation may be more complex than that of the mantle epithelium.

Previous studies have described the deposition of the periostracum on the nucleus just after implantation, followed by the prismatic layer and then the pearl layer [1]. After implantation, the expression pattern of the matrix genes in the pearl sac epithelium may switch from that forming the prismatic layer to that forming the nacreous layer [31]. It is likely that, in the prismatic pearl, this transition from prismatic-specific genes to nacreous-specific genes was disturbed by unknown substances, perhaps originating from aggregates of dead cells derived from gametes or hemocytes interposed between the pearl and the pearl sac [3]. Enhanced expression of prismatic layer forming genes may then continue, leading to the deposition of the thick prismatic layer. Further clarification of the process of this transition in the pearl sac is important for the development of methods to improve pearl quality.

## Materials and Methods

### Juveniles

To observe the expression pattern of whole mantle tissue, three-months-old juveniles of *Pinctada fucata* (9–10 mm in shell height) were fixed with 4% paraformaldehyde (PA) in 0.2 M PBS (pH 7.2) for *in situ* hybridization (ISH). We selected five healthy oysters uninfested by parasites, such as *Polydra* species. After fixation, the shells were removed and whole soft tissue was processed for paraffin embedding.

The juveniles were produced in the hatchery of Mie Prefecture Fisheries Laboratory and were attached to a raft in Ago Bay, Mie Prefecture. No permissions were required because the specimens were artificially produced. This species is not endangered or protected.

### Implantation experiment

**Figure 9:**
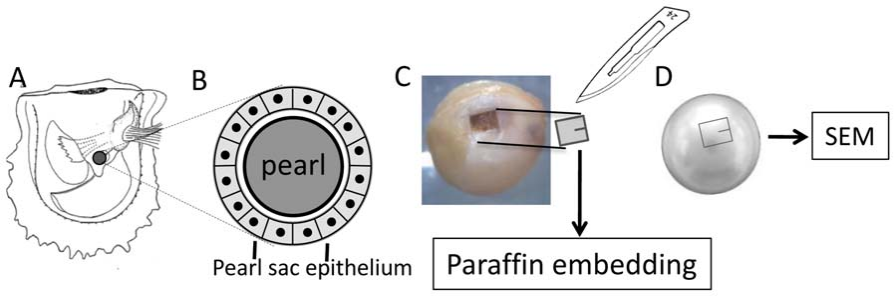
Schematic drawing and photograph of the region of the oyster including the pearl sac. (A) The position of the pearl sac formed in the distal region of the oyster. (B) Schematic drawing of a vertical section of the pearl sac epithelium surrounding the pearl. (C) Dissection of the pearl sac tissue from the whole pearl sac for ISH. (D) After dissection of the pearl sac samples for ISH, we scratched the surface of pearl with a scalpel as a reference for SEM observation.

Host oysters (mean hinge length 50.8 mm) were ‘pre-operative conditioned’ for two weeks before the implantation [32]. Round nuclei (8 mm diameter) made from freshwater mussel shell were purchased from a supplier. A nucleus and a small piece of mantle tissue (2–3 mm2) dissected from other oysters (mantle donors) were inserted into the ‘pearl pocket’ in the distal region of the visceral mass (Fig. 9A) of the mother (host) oysters in June 2009. After implantation, the oysters were transferred to panel nets and hung from a raft in Ago Bay. The whole soft parts were fixed with 4% PA in 0.2 M PBS for ISH 38 days after implantation. No permissions were required because oysters were purchased from private farmers and all experiments were conducted in the facilities of Mie Prefecture Fisheries Laboratory and Mie University.

### Mantle graft and pearl sac fixation for *in situ* hybridization

During nucleus implantation, mantle tissue for grafting was dissected from donor oysters, cut into small pieces (2–3 mm^2^), implanted into host oysters by a skilled technician, and fixed with 4% PA in 0.2 M PBS (pH 7.2) at 4°C, for ISH.

Thirty-eight days after implantation, the shells were removed and whole soft parts of implanted oysters were fixed with 4% PA for at least 48 h at 4°C. The fixed tissue was then transferred to 90% ethanol and the pearl and pearl sac in the distal part of the body (Fig. 9A, B) was dissected out under a stereomicroscope using forceps and scissors. The small piece of pearl sac tissue was excised with a scalpel (Fig. 9C). A scratch was made on the pearl surface as a reference for the area analyzed by ISH (Fig. 9D) and the pearls were harvested. The area of the pearl sac used for ISH is shown in Figs. 4A, B and 6A. The dissected tissue was processed for dehydration using an ethanol series and conventional paraffin embedding.

### Pearl quality grading

The quality of pearls obtained from nine oysters was determined using the naked eye and a tabletop scanning electron microscope (TM-1000, Hitachi High-Technologies Corporation, Tokyo, Japan). The pearls were observed by scanning electron microscope without coating.

### *In situ* hybridization (ISH)

The sequences of MSI31 and MSI60 (GenBank accession numbers D86073 and D86074) were used to design a probe for ISH and prepared using a DIG RNA labeling kit with T7 RNA polymerase (Roche, Indianapolis, IN, USA). ISH was performed according to the method described [33] and slightly modified [34]. Proteinase K treatment (1 µg/mL) was carried out for 15 min at 37°C. Hybridization was carried out at 65°C overnight. Blocking was performed with Blocking Reagent (Roche) before the antibody reaction. A mixture of BCIP/NBT was used for color development of the anti-Digoxigenin-AP Fab fragments (Roche). After ISH, each sample was counterstained by Nuclear Fast Red (VECTOR, Burlingame, CA, USA) or eosin and observed under a light microscope (E600, Nikon, Tokyo, Japan).
